# Plant Oils in Sport Nutrition: A Narrative Literature Review

**DOI:** 10.3390/nu17243943

**Published:** 2025-12-17

**Authors:** Kinga Kostrakiewicz-Gierałt

**Affiliations:** Department of Tourism Geography and Ecology, Institute of Tourism, Faculty of Tourism and Recreation, University of Physical Education in Kraków, Jana Pawła II 78, 31-571 Krakow, Poland; kinga.kostrakiewicz@awf.krakow.pl

**Keywords:** fatty acids, health, patent, performance, plant, recovery

## Abstract

**Background/Objectives.** Edible oils derived from herbaceous and woody plants are an important nutritional resource, assuring the health and performance of sportspeople. The aim of this study was to review the inventions and experimental articles referring to the application of vegetable oils in food products for sportspeople and published in the period of 2015–2024. **Methods**. The literature search was conducted across Google Scholar, Scopus, and ISI Web of Science databases, as well as by using Google Patents and Espacenet Patent search engines. **Results**. Altogether, 58 patents and 35 original articles were found. In total, the use of 39 plant taxa belonging to 27 botanical families was documented. The majority of disclosures refer to sports nutrition, post-exercise recovery support, and/or sport performance improvement and may be provided in the form of powders, tablets, beverages, and/or capsules. According to the reviewed studies, the consumption of olive, walnut, and perilla oils beneficially affects the morphological, physiological, and biochemical indicators of sportspeople. The substantial intake of olive oil reported by sportspeople from southern Europe is linked to the recommendations of the Mediterranean diet, while lower consumption of other vegetable oils might be connected to focusing on intake of carbohydrates and/or proteins and/or consumption of other fat sources such as seeds or nuts. **Conclusions**. Considering the great potential of useful plant species, it might be concluded that future investigations should focus on both (i) further investigations of the effects of well-known vegetable oils on the health and performance of sportspeople, and (ii) searching for novel plant oil sources, suitable for the preparation of food products dedicated to amateur and professional sportspeople.

## 1. Introduction

Edible oils derived from both animals (e.g., marine fish and shellfish) and selected herbaceous and woody plants are important and indispensable nutritional resources for human health. Although the chemical composition of particular oils might differ, their main components (usually present at 94–99% of the total lipid amounts) are triacylglycerols (TAGs) formed by the esterification of glycerol with three fatty acids. The fatty acids are classified into saturated fatty acids (SFAs), containing no double bonds within the hydrocarbon chain, and unsaturated fatty acids (UFAs), represented by monounsaturated fatty acids (MUFAs) with a single carbon–carbon double bond and polyunsaturated fatty acids (PUFAs) with at least two carbon–carbon double bonds. The chemical structure of UFAs is described by the omega (ω) system based on the position of the first carbon–carbon double bond counting from the methyl end of the hydrocarbon chain. In the case of *n*-9 MUFA, only one double bond is located at carbon 9 of the fatty acid molecule, whereas in the case of *n*-6 and *n*-3 PUFA, the first double bond is at carbon 6 and 3, respectively. The human body can synthesize the required SFAs and ω-9 fatty acids, including, among others, oleic acid (OA). Fatty acids containing two or more double bonds must be obtained from the diet, and, therefore, the latter are called essential fatty acids. The chief ω-6 fatty acid is represented by linoleic acid (LA), while the most important ω-3 fatty acids are linolenic acid (ALA), followed by eicosapentaenoic acid (EPA) and docosahexaenoic acid (DHA).

At the same time, it is worth mentioning that LA and ALA are found in plants, while EPA and DHA are found in fish oils. According to many authors (e.g., [1,2,3,4,5,6]), unsaturated fatty acids play important roles in the human body, such as maintaining the relative fluidity and flexibility of cell membranes to ensure the normal physiological function of cells, esterifying cholesterol, and reducing cholesterol and triglyceride in the blood. The intake of ω-6 fatty acids lowers the risk of cardiovascular diseases, while the consumption of ω-3 fatty acids diminishes the risk of metabolic syndrome and chronic diseases characterized by elevated inflammation and benefits muscle and cognitive performance, as well as having potential use in COVID-19 therapy [7]. At the same time, it should be pointed out that the balance between omega-3 and omega-6 fatty acids is crucial for health because both types compete for the same enzymes in the body. Although recommendations vary, it is generally accepted that the ideal ratio of ω-6 and ω-3 fatty acids should be between 2:1 and 4/5:1 [8,9].

Apart from triacylglycerols and free fatty acids, edible plant and fish oils contain secondary products, among others, polyphenols, tocopherols, and carotenoids. As stated by several authors (e.g., [10,11,12,13,14]), these increase the shelf-life of oils, reportedly reduce cardiovascular disease, show liver and kidney protective effects, and provide some anti-obesity, antioxidant, and anti-inflammatory properties. Edible oils of both plant and animal origin also contain trace metals present in small amounts and represented, among others, by essential nutrients such as iron (Fe), copper (Cu), and zinc (Zn), whereas others are considered toxic, such as arsenic (As), lead (Pb), cadmium (Cd), and chromium (Cr) [13,15,16,17].

The extraction of vegetable oils has a long history. As reported by many authors (e.g., [13]), in early human history, sesame (*Sesamum indicum*) oil and olive (*Olea europaea*) oil were commonly used. Much later, in the East, soybean oil may have been extracted for culinary purposes. Nowadays, the global production of vegetable oils has seen a steady increase since the beginning of the century, reaching a peak of 229 million metric tons in 2024/2025 [18]. Today, in the case of many plants, the composition and content of bioactive components, as well as the preparation, refining, authenticity identification, and applications of edible vegetable oils, are well-known [19,20]. Nevertheless, the search for other promising edible oil sources is still being conducted (e.g., [21,22,23,24]). The aforementioned publications provide evidence that good oil sources include the seeds of the melon *Cucumis melo* and peony *Paeonia* sp., as well as the stems and leaves of the sugar cane *Saccharum officinarum* and sweet sorghum *Sorghum bicolor*. At the same time, Zhou et al. [24] argued that despite many challenges, significant development in engineering nutritionally improved edible plant oils has recently been achieved. The increase in the world population and the desire to consume healthier food will drive ever-escalating demand for nutritionally improved edible plant oils.

The favorable impact of plant oils on human health has been repeatedly pointed out, among others, in the case of avocado *Persea americana* Mill [25], rapeseed *Brassica napus* [7,26,27], coconut *Cocos nucifera* [27,28], corn *Zea mays* [29], flax *Linum usitatissimum* [7,27,30,31], horseradish tree *Moringa oleifera* [32], olive *Olea europaea* [7,27,33,34,35,36], sesame *Sesamum indicum* L. [37], and soybean *Glycine max* [7,38]. The main health benefits are represented by improvement of cardiovascular health (e.g., [25,26,34,36,37,38]), prevention of metabolic syndrome symptoms (e.g., [25,26,36]), relief of memory disorders (e.g., [37]), and increase in anti-inflammatory (e.g., [25,28,31,32,33,34,37]), antioxidant (e.g., [25,28,31,32,33,34,37]), anticancer (e.g., [28,31,33,34,35,36]), antiaging (e.g., [30,37]), and antiallergic (e.g., [33]) activities. At the same time, Trivedi et al. [39] pointed out the beneficial effects of plant oils on different aspects of sport performance. They reported that intake of almond, macadamia, and hazelnut oils contributes to an increase in athletic endurance, while consumption of walnut and pistachio oils accelerates post-exercise recovery. The aforementioned authors also stated that intake of almond and hazelnut oils reduces muscle damage, whereas walnut oil positively influences joint health. The strongly recommended consumption of plant oils for athletes [40,41] was particularly relevant for optimal maintenance of physical and mental fitness during the restrictive second wave of the COVID-19 confinement period [42]. Surprisingly, the investigations of the impact of the consumption of plant oils on sport performance have been rather rarely summarized (see e.g., [43,44]). Philpott et al. [43] reported selected outcomes regarding the influence of olive oil and corn oil consumption on recovery following exercise-induced muscle damage. In turn, Perrone and d’Angelo (2025) [44] focused on the role of extra-virgin olive oil intake in mitigating oxidative stress and inflammation. Thus, considering the insufficient current state of knowledge, the presented investigations were undertaken, and the specific goals aimed to answer the following questions:

1. Which plant taxa are sources of oils in patented alimentary products dedicated to sportspeople?
2. What is the consistency, form, and activity of the above-mentioned products?
3. What is the impact of plant oils on the performance and health of sportspeople?
4. What is the frequency of use of plant oils by sportspeople?

## 2. Materials and Methods

### 2.1. Literature Search

Scientific articles were searched for by browsing the ISI Web of Science (all databases), Scopus, and Google Scholar (the most widely used database for bibliometric analyses), while patents were searched for by browsing the Google Patents and Espacenet Patent search engines, gathering the largest number of open-access patents [45,46]. The survey of literature records published from 1 January 2015 up to 31 December 2024 was carried out with factorial combinations of the following keywords in the searches: (‘oil’) and (’plant’ or ‘vegetable’) and (‘sport’ or ‘athlete’). The selection terms were examined from the title and abstract. The literature search was conducted from 1 to 31 August 2025. Due to the number of records ranging from a dozen to tens of thousands, after searching for particular combinations of keywords, the analysis was limited to the first 500 records in the case of Google Scholar, Google Patents, and Espacenet. Such a number was judged to be appropriate after a pilot study showing that, with increasing record numbers over 500, the number of duplicates increased substantially. As such, the search included 2000 hits from Google Scholar, 2000 from Google Patents, and 2000 from Espacenet. Following the removal of duplicates (publications indexed in more than one database), the abstracts and descriptions of patents were screened for relevance and eligibility.

### 2.2. Study Eligibility and Selection

During screening of the abstracts and descriptions of patents, the inclusion criteria were as follows: (i) the invention refers to the use of plant oils in sport nutrition, (ii) the description of the invention contains specification of the plant taxa used as the raw material, (iii) the patented alimentary items are suitable for human consumption, and (iv) the abstract and description of the patent are written in English. The exclusion criteria were as follows: (i) the invention is not relevant to the main topic of the review (e.g., the product is for external use, e.g., creams, or the product does not contain specified plant taxa names), (ii) the source of oils does not belong to the plant kingdom, (iii) the invention is intended for animal consumption, (iv) the abstract and description of the patent are not written in English, and (v) the abstract and description of the patent are absent.

**Figure 1:**
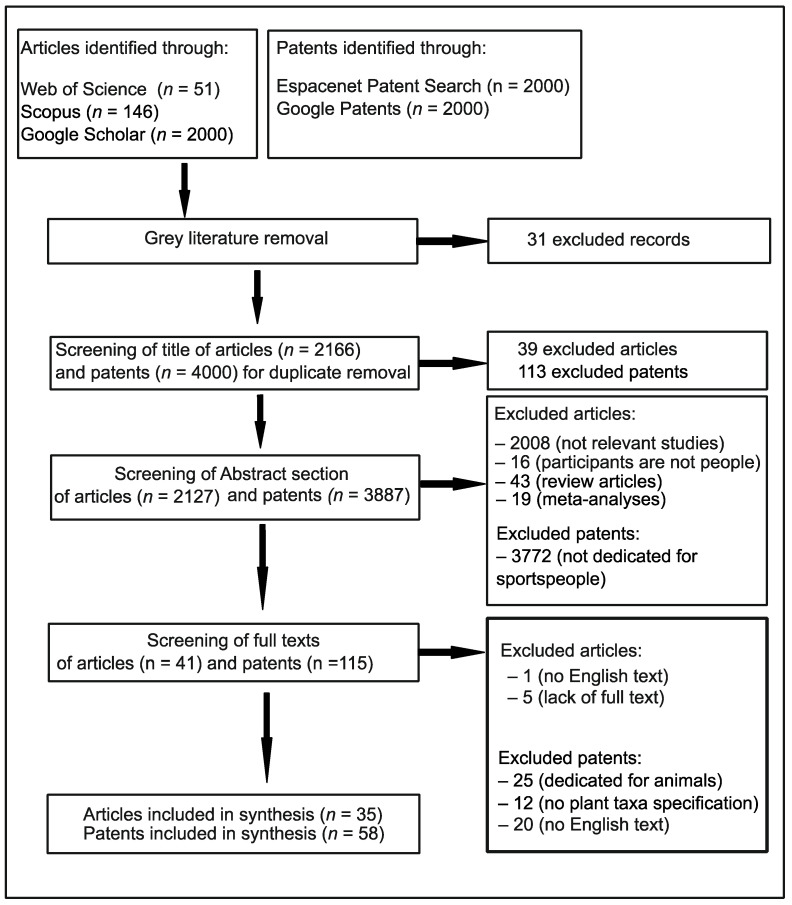
The procedure for the patent and article search.

During screening of the abstracts and full-texts of articles, the inclusion criteria were as follows: (i) the investigations are relevant to the application of plant-based oils in sport nutrition, (ii) the investigations included humans as participants, (iii) the investigations were observational, descriptive studies (case report/case series), observational, analytical studies (case–control studies, cross-sectional studies, and cohort studies), or experimental studies (randomized controlled trials), (iv) there were no limits regarding the age, weight, sex, nationality, or number of participants, (v) there were no limits in geographical location or time period of the investigations, and (vi) the abstract and full text are written in English. The exclusion criteria for articles were as follows: (i) there is no available full-text version, (ii) the investigations are meta-analyses or systematic reviews due to the assumption that the present review is based on primary, empirical studies. A chart detailing the search results is presented in Figure 1.

Additionally, it should be pointed out that although the procedure of literature identification and screening was consistent with PRISMA statements, the eligible publications became the basis for preparing a narrative review with strong descriptive aims and some limitations addressed in the Discussion Section. To assess the quality of the included studies and reduce the potential for misclassification, the abstracts and then descriptions of patents were subjected to critical double screening. Furthermore, in the case of publications based on investigations on humans, the bias risk was assessed. The following risks of bias were evaluated: (i) bias arising from the randomization process; (ii) bias due to deviations from intended interventions; (iii) bias due to missing outcome data; (iv) bias in the measurement of the outcome; and (v) bias in the selection of the reported result. Each paper was evaluated with the criteria and judged as “low bias risk” (denoted as “L”) if the criteria mentioned were fulfilled, “high bias risk” (denoted as “H”) if otherwise, and “some concerns” (denoted as “SC”) if some criteria were not fulfilled. The following data were extracted from eligible patents: author names, year of publication, title of patent, and characteristics of the invention (consistency, form, and plant species used as a source of oils). The scientific and common names of particular taxa, the biome of natural occurrence, and the lifespan and life form were checked using the Plants of the World Online database [47]. The aforementioned data were extracted using a form created in Microsoft Excel.

## 3. Results

### 3.1. Plant Taxa as Sources of Oils Used in Sports Food and Drinks

The analysis of patented inventions [48,49,50,51,52,53,54,55,56,57,58,59,60,61,62,63,64,65,66,67,68,69,70,71,72,73,74,75,76,77,78,79,80,81,82,83,84,85,86,87,88,89,90,91,92,93,94,95,96,97,98,99,100,101,102,103,104,105] (Table A1) documented the use of 39 plant taxa belonging to 27 botanical families. The greatest number of plants used as an oil source was represented by the family *Brassicaceae*, followed by the *Juglandaceae* and *Poaceae* families. The species mostly mentioned in patented inventions included *Olea europaea* L., *Glycine max* (L.) Merr., *Cocos nucifera* L., *Brassica napus* L., *Helianthus annuus* L., *Arachis hypogaea* L., and *Zea mays* L. Meanwhile, the taxa mentioned solely in one patent description were represented by *Anacardium occidentale* L., *Bertholletia excelsa* Humb. & Bonpl., *Brassica juncea* (L.) Czern., *Brassica oleracea* var. *italica* Plenck, *Camelina sativa* (L.) Crantz, *Carya illinoinensis* (Wangenh.) Koch, *Citrullus lanatus* (Thunb.) Matsum. & Nakai, *Coriandrum sativum* L., *Corylus avellana* L., *Cucurbita pepo* L., *Fagus sylvatica* L., *Hippophae rhamnoides* L., *Juglans major* (Torr.) A. Heller, *Nigella sativa* L., *Salvia hispanica* L., *Sinapis alba* L., and *Triticum aestivum* L. (Table 1).

**Figure 2:**
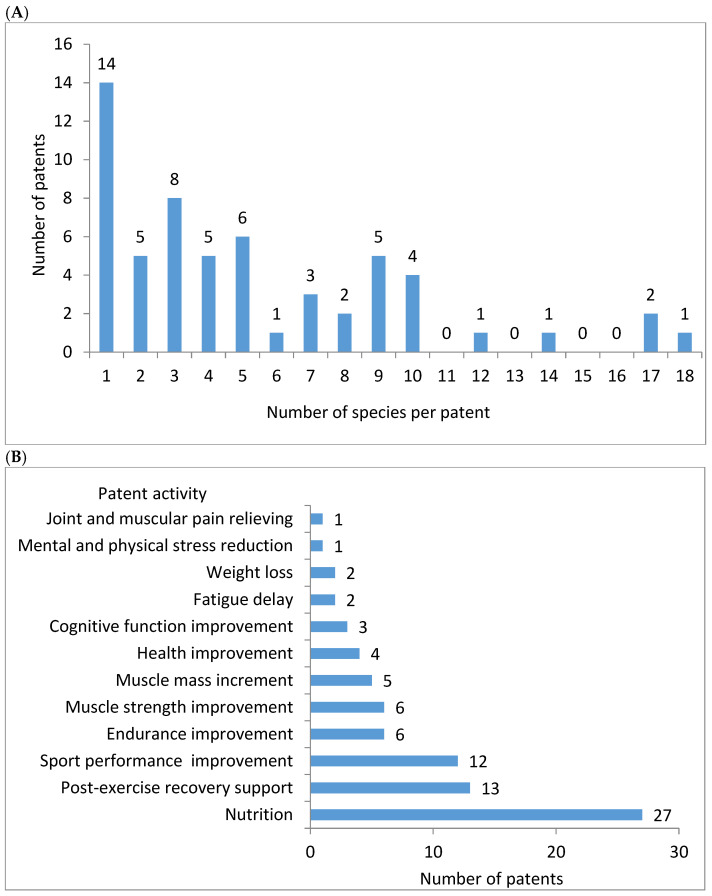
The number of plant species per patent (**A**) and the activities of products (**B**) mentioned in the descriptions of inventions referring to sports nutrition and using plant species as an oil source published in the years 2015–2024.

From 1 to 18 species per patent were found in the surveyed description of patented inventions (Figure 2A). The greatest number of patents was based on one plant species as the source of oil. From 2 to 10 species were mentioned in a lower number of inventions, while more than 10 species were mentioned only in one or two inventions. The performed survey evidenced that the patented products focused on a wide area of activities (Figure 2B). The majority of disclosures referred to the nutrition of sportspeople. A considerable number of patents concentrated on post-exercise recovery support, as well as sport performance improvement. Other areas of activity were linked to improvement of athletic endurance, as well as muscle mass and strength. Relatively few authors pointed out the role of the inventions in the improvement of health or cognitive function, delay of fatigue, weight loss, mental and physical stress reduction, or relief of joint and muscular pain.

**Figure 3:**
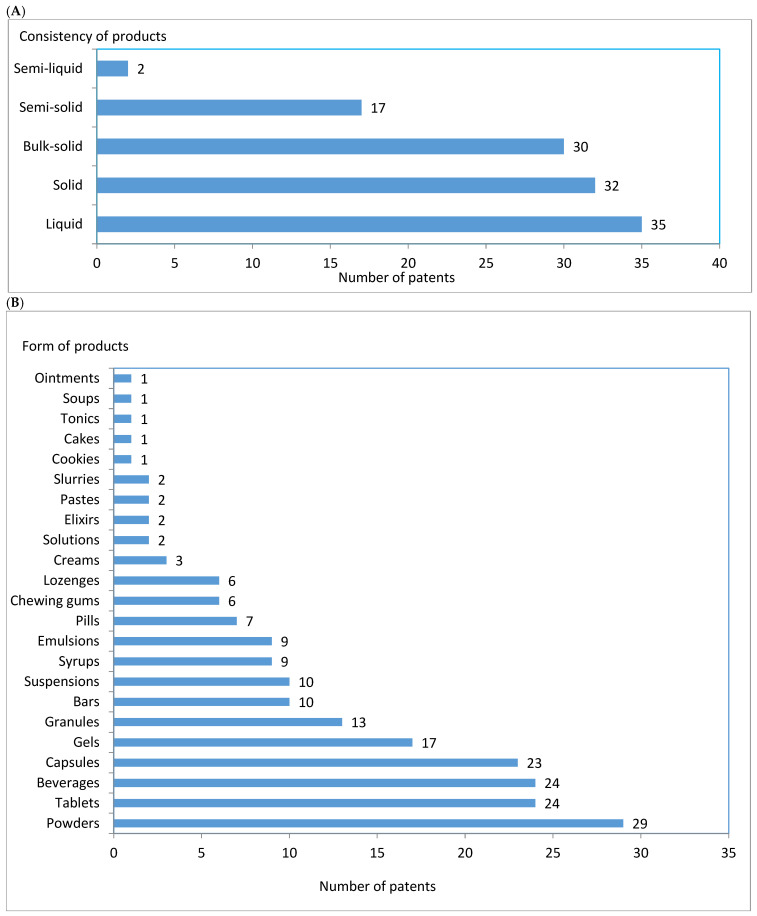
The consistency (**A**) and form (**B**) of products mentioned in the descriptions of inventions referring to sports nutrition and using plant species as an oil source published in the years 2015–2024.

According to the patent description, the invented products might be provided in a variety of consistencies: from liquid, solid, bulk-solid, and semi-solid to semi-liquid (Figure 3A). Altogether, 23 different forms of products were mentioned in the surveyed patent descriptions. From 23 to 29 products might be provided in the form of powders, tablets, beverages, and/or capsules. From 3 to 17 products might be provided in the form of gels, granules, bars, suspensions, syrups, emulsions, pills, chewing gum, lozenges, and/or creams. Other forms, such as solutions, elixirs, pastes, slurries, biscuits, cakes, tonics, soups, and ointments, were only rarely mentioned in the patent descriptions (Figure 3B).

### 3.2. The Impact of Plant Oils on the Performance and Health of Sportspeople

The majority of authors focused on the effects of olive oil on the health and performance of sportspeople (Table 2). Capó et al. [106,107] analyzed the effects of an almond-based beverage enriched with olive oil, exercise, and age on inflammatory plasma markers and immune gene expression in peripheral blood mononuclear cells (PBMCs) in young sport practitioners and senior athletes. They evidenced that the supplementation of a functional beverage among young athletes enhances a pro-inflammatory circulatory environment in response to exercise [106] but protects against oxidative damage after exercise [107]. Esquius et al. [108] noted that supplementation with extra-virgin olive oil increased the cardiorespiratory system during a progressive walking test at moderate intensity, although it did not change performance or other physiological markers. Esquius et al. [109] showed that extra-virgin olive oil supplementation can reduce the inflammatory impact of intense aerobic effort and improve recovery at 24 h. Mielgo-Ayuso et al. [110] investigated the associations between exercise-induced muscle damage (EIMD) and exercise-induced cardiac stress (EICS), and endurance athlete diets one week before a marathon race. They evidenced the positive effect of the consumption of olive oil on cardiac muscle health. Mieszkowski et al. [111] evidenced that ultramarathon running induces the mobilization of vitamin D into the blood. Furthermore, Mieszkowski et al. [112] demonstrated that the inflammatory response induced by ultramarathon running is significantly blunted in runners who received a single high dose of vitamin D in a solution of vegetable oils before the run. Additionally, Ayari and Boukazoula [113] observed that virgin olive oil supplementation can improve hormonal status in half-marathon athletes.

Moreover, Kamoun et al. [115] documented that moderate walnut consumption improved lipid profiles, steroid hormones, and inflammation in recreationally training elderly men. Sinaga [118] evidenced that red fruit oil consumption increases the levels of hemoglobin, erythrocytes, and hematocrit in athletes during maximal physical activity. The authors also argued that the addition of red fruit oil to a training program can increase the endurance of athletes during maximal physical activity. Meanwhile, Nieman et al. [117] stated that ingestion of chia seed oil cannot be recommended as an ergogenic aid during intensive, prolonged running or as a countermeasure to exercise-induced inflammation. Also, Borba et al. [114] documented that intake of coconut oil does not improve running times, nor influence the rating of perceived effort and lactate concentrations in recreational runners. In turn, Kawamura et al. [116] found that the intake of perilla oil improves gut function and enhances the abundance of the butyrate-producing bacteria Lachnospiraceae and suppresses urinary indoxyl sulfate levels in trained athletes, whereas Tang et al. [119] evidenced that the administration of dietary flaxseed oil enhanced the prognosis of acute anterior cruciate ligament rupture in sportspeople.

The risk of bias of publications presented in human studies is given in Table 3. Due to the fact that the majority of investigations showed a low risk of bias, the overall rating for all studies across all categories is “low risk”.

Moreover, several methodological limitations in the reviewed investigations were presented. Some limitations were connected to the participants of the investigations, and the occurrence of an insufficient number of participants was noted by Borba et al. (2019) [114], Capó et al. (2016) [106], Esquius et al. (2021) [109], and Mielgo-Ayuso et al. (2020) [110], while the lack of a control group was noted by Kamoun et al. (2021) [115] and Tang et al. (2022) [119], and only one sex of participants was noted by Esquius et al. (2021) [109]. Other limitations were linked to the measurements and/or biochemical analyses. A lack of daily surveys throughout the intervention period due to the inconvenience for the participants was claimed by Kawamura et al. (2023) [116]. Furthermore, they argued that a limitation of the study was the unequal number of interventions among groups. Group 1 received the intervention three times daily to reach the targeted dose, while the other two groups received it once daily. The aforementioned authors added that another limitation was the lack of a direct measure of the changes in short-chain fatty acid levels. In the case of investigations of the effect of acute caffeine and coconut oil intake on running time (Borba et al. 2019 [114]), the inability to measure the blood caffeine level and its metabolite concentrations was highlighted as the main limitation. Esquius et al. (2019) [108] stressed the lack of continuous blood pressure monitoring and the use of biomarkers to observe the physiological oxidative status of participants, as well as the low frequency of monitoring of oxygen and CO2 content and air flow rate. Moreover, the aforementioned authors stated that due to a lack of assessment of inflammatory and lipid peroxidation markers, it cannot be guaranteed that the changes observed in cardiorespiratory coordination under olive oil supplementation were provoked by physiological adjustments at this level. Mielgo-Ayuso et al. (2020) [110] stated that a limitation of the studies was the lack of demonstrated pre-competition values of the exercise-induced muscle damage (EIMD) and exercise-induced cardiac stress (EICS) parameters. Moreover, the authors stated that the results of this investigation might be applicable to runners with a Mediterranean-based diet, while the relationship between food intake and EIMD and EICS should be studied in other cultures and racial groups. In the case of investigations regarding the effect of dietary flaxseed oil on the prognosis of acute anterior cruciate ligament rupture, Tang et al. (2022) [119] stated that one methodological limitation might be connected to the fact that reconstructive and surgical procedures might also induce inflammation. Other limitations were connected to outdoor conditions. The lack of measurements of the wind speed (influencing running performance) during the marathon race was pointed out by Borba et al. (2019) [114]. In the case of the remaining investigations (Ayari and Boukazoula (2023) [113], Capó et al. (2016b) [107], Mieszkowski et al. (2020) [111], Mieszkowski et al. (2021) [112], Nieman et al. (2015) [117], and Sinaga (2017) [118]), the limitations were not stated.

### 3.3. The Frequency of Use and Sensory Acceptability of Food Products Containing Plant Oils by Sportspeople

The majority of authors studied adherence to the Mediterranean diet also in the context of the use of olive oil (Table 4). Muros and Zabala [120], Kontele et al. [121], Santos-Sánchez et al. [122], and Leão et al. [123] documented the substantial use of olive oil by sportspeople in Portugal, Greece, and Spain. In particular, Vélez-Alcázar et al. [124] confirmed that the majority of adolescent athletes showing an excellent or moderate adherence to the Mediterranean diet reported the consumption of olive oil at home. Furthermore, other authors documented that adherence to the Mediterranean diet and use of olive oil at home are positively correlated with levels of nutritional education [125], as well as levels of physical activity [126]. In turn, other authors noted the lower consumption of olive oil by sportspeople in Saudi Arabia [127,128] and Poland [129,130]. Numerous authors focused on comparisons of the use of olive oil among demographic groups. Santana et al. [131] confirmed considerable intake of olive oil by younger and adolescent female rhythmic gymnasts. Muros et al. [132] evidenced that females tend to consume more olive oil than males, whilst triathletes consume more olive oil than cyclists. Other authors found greater consumption of olive oil by senior sportspeople than younger ones [133], by professional athletes than amateur ones [134], as well as by adolescent sportspeople than by adolescent people not practicing any sport [135].

At the same time, many authors studied the consumption of other vegetable oils. Szot et al. [130] recorded that canola oil is ingested once or twice a week, while coconut oil is consumed less often by Polish esports players. The investigations of Ritz et al. [138] were concentrated on the sources of omega-3 fatty acids in the diets of athletes. The obtained results confirmed that canola oil is the most frequently consumed plant source of alpha-linolenic acid. Additionally, Hooks et al. [136] demonstrated that canola oil is consumed by numerous athletes as opposed to flaxseed oil. Ventura Comes et al. [140] investigated the consumption of nutritional supplements by squash players at international vs. national levels. The aforementioned authors found that consumption of flaxseed and coconut oils is greater in the group of international players. Staśkiewicz et al. [139] observed that raw vegetable oils, unincorporated into meals, are eaten 3–4 times a week, and the frequency of their consumption did not differ among amateur and professional sportspeople. Moreover, the aforementioned authors recorded that the intake of vegetable oils did not vary among practitioners of CrossFit, bodybuilding, football, or handball. In addition, Novokshanova [137] confirmed the use of vegetable oils as a fat source.

The main methodological limitation of the aforementioned studies was the cross-sectional design of the studies (offering only a static view of the population at a given time, and not addressing the causality of the results) [120,121,124,126,128,132,133,134,135]. Another limitation was caused by the use of self-reporting questionnaires, which pose the risk of socially desirable answers or memory bias [120,121,122,123,126,130,132,134]. Other authors stated that some biases were connected to a limited sample size [124,127,133,135,136,139] and the concentration of the study in a solitary locale [127]. Numerous limitations were connected to the young age of participants and the fact that investigations were conducted only in one city [126]. Santos-Sánchez et al. [122] pointed out the lack of data regarding the maturity level and the sport performance of participants, while other authors [130,132,135] underlined the unequal participation of women and men or participation of one sex of participants, which might mask gender differences. Martinovic et al. [134] indicated the significant differences in the education level of the included participants, which may have possibly influenced the results. Some researchers highlighted that the different number of investigation participants practicing a particular sport discipline might make it difficult to make sport-by-sport comparisons [138]. In turn, the others argued that the limitation of the studies was the lack of representativeness of players from other clubs and other sports disciplines [139]. Several authors pointed out that a potential source of bias might be the lack of precise measurements of the anthropometric characteristics of the athletes (height and weight), which were self-reported by participants [120,121,128] or the absence of data on anthropometric parameters [138]. Furthermore, some limitations were connected to the impossibility to acquire additional data (such as body fat, muscle mass, waist circumference, and sexual maturation) [121,123,138] and daily physical activity, or more direct measures of cognitive performance reflecting brain function [130], to estimate calorie and nutrient intake [122,124,128,133], as well as to analyze actual food patterns [123]. Some authors admitted that limitations might be caused by the inclusion in the investigations of individuals with chronic illnesses or those taking medications [128]. Martínez-Rodríguez [133] stated that one limitation might be the lack of studies to compare the obtained results with [133]. Additionally, it should be pointed out that limitations were not reported in the studies by Gacek and Frączek [129], Novokshanova [137], Philippou et al. [125], Santana et al. [131], or Ventura Comes et al. [140].

## 4. Discussion

The observed greater number of patented alimentary products for sportspeople containing plant oils provided in liquid and solid consistency than in semi-solid and semi-liquid ones seems to support the trend noted by Kostrakiewicz-Gierałt [141], who surveyed the food products dedicated to sportspeople based on plant proteins, peptides, and amino acids. However, the greatest number of products containing vegetable oils, which can be provided as powders (bulk-solid consistency), corresponds with the popularity of this form of food and supplementation among amateur and professional sportspeople (e.g., [142,143,144]). Moreover, many patented products can be provided in the frequently used form of tablets, beverages, and/or capsules, which are widely appreciated by athletes.

The investigations conducted showed that the greatest number of plants used as an oil source in patented inventions was represented by the family *Brassicaceae*. Such an outcome is not surprising, considering the large number of these economically important plants of value for food and medicinal purposes. Some of these are commercially cultivated as oilseeds to meet the global demand for a healthy plant-derived oil, high in polyunsaturated fats, i.e., *B. napus* and *B. juncea*. Other species are foraged from the wild, i.e., *E. repandum* and *S. erysimoides*, and harvested for medicinal uses. These plants contain a diverse range of bioactive natural products, including sulfur-containing glucosinolates and other potentially valuable compounds, namely, omega-3-fatty acids, terpenoids, phenylpropanoids, flavonoids, tannins, S-methyl cysteine sulfoxide, and trace elements [145]. Gidik and Öneml (2023) [146] compared the oil content in selected species and argued that the highest oil content was determined in rapeseed. In addition, wild mustard and black mustard were found to contain higher amounts of oil than white mustard. Moreover, the highest values of oleic and linoleic acids were found in rapeseed and of linolenic acid in false flax, while erucic acid was highest in wild mustard. Simultaneously, it should be added that erucic acid shows both toxic and beneficial properties [147]. Considering the numerous beneficial properties of taxa representing the *Brassicaceae* family, the use of a few species in patented inventions seems to be intriguing. At the same time, it can be stated that the scarce use of species from other families demonstrated in the presented review might be connected to underutilization of plants as an oil source. Such a phenomenon was evidenced for the *Fabaceae* family.

The performed survey of patent documents showing that the majority of patented products are dedicated to sports nutrition corresponds with the findings of Khalili Tilami and Kouřimská [148], which evidenced the high dietary quality of plant lipids mentioned in the patent descriptions. Moreover, the taxa most frequently mentioned in the patented inventions as a vegetable source of oils were also used as a lipid source in plant-based meat analogs [146,149]. At the same time, it should be pointed out that the most frequent mentions in patent descriptions and substantial world production seem to remain in accordance, in the cases of oil derived from olive [147,150], soybean [148,151], coconut [149,152], canola [150,153], sunflower [151,154], and peanut [152,155]. The most frequent use of oils derived from the aforementioned species might be linked to new methods of their improvement. Thanks to modern methods, the seed oils of the aforementioned plants might be biochemically modified to increase the content of desired components or reduce the content of undesirable components [24]. In turn, advanced oil extraction methods ensure extraction of superior-quality oil and do not have any harmful impact on the environment [154]. However, according to Akmeemana et al. [156], the demand for vegetable oils exceeds the global population growth rate. Consequently, alternative, underutilized plant sources must penetrate the market to fulfill future demand. The aforementioned authors stated that plant oils derived from other (also rarely or not mentioned in the reviewed inventions) sources, such as lotus, moringa, black cumin, avocado, soursop, tamarind, and many others, provide a variety of health benefits.

The use of olive oil in patented inventions is not surprising considering its positive effects on human health, spanning from the prevention of cardiometabolic disorders and cancers to neuroprotective properties, among others, thanks to a substantial content of polyphenols [153,154,155,156,157,158,159,160,161,162] and ω-9 oleic acid [153,158]. Other rich sources of ω-9 oleic acids include, among others, rapeseed, maize, and soybean oils [158,163]. Nevertheless, as regards PUFA, it is worth mentioning that from the aforementioned species, only rapeseed oil shows the optimal ratio for human health of ω-6 and ω-3 fatty acids, reaching 2:1 [159,164]. In the case of the other oils, the aforementioned ratio is exceeded and ranges from 11.7:1 in olive, 25.8:1 in peanut, and 71.6:1 in maize to 221.3:1 in sunflower oil [9]. Meanwhile, surprisingly, oils from black cumin, hemp, flax, and walnut, which also present the optimal ratio—achieving, respectively, 2:1, 3.3:1, 3.5:1, and 4.8:1—were rather rarely mentioned in the reviewed patent descriptions. Apart from ω-fatty acids, the plants mentioned in the patent descriptions contain many other beneficial compounds, such as α-, β-, and δ-tocopherols [10,11], polyphenols (as well as flavonoids such as proanthocyanidins), carotenoids, phytosterols, and squalene [10]. The aforementioned authors argued that a much higher polyphenol content is presented by olive oil than by macadamia, avocado, sesame, canola, soy, grapeseed, sunflower, walnut, peanut, or almond oils. Squalene, present inter alia in olive oil, is a precursor of sterols [10]. Phytosterols such as β-sitosterol are present in avocado, olive, hazelnut, and grapeseed oils, while δ(5)-avenasterol occurs in olive and hazelnut oils.

Despite the considerable number of oil-bearing species, the effects on sportspeople’s health and performance were evidenced solely in the case of consumption of olive, walnut, and perilla oils. Such a phenomenon is caused by the lack of investigations or the limitations of the reviewed investigations regarding participants (e.g., small sample size or lack of a control group) or measurements and biochemical analyses, which could have had an influence on the obtained results. The performed studies confirmed that the intake of these beneficially affects morphological (e.g., blood cell number and levels of hemoglobin and hematocrit), physiological (e.g., VO2 max. and ventilation efficiency), and biochemical (e.g., glucose, triglyceride, hormone, and enzyme levels in the blood) indicators, similar to the consumption of animal oils reported, among others, by Philpott et al. [43], D’Angelo et al. [160,165], Lewis et al. [161,166], Thielecke and Blannin [162,167], and Shaw et al. [163,168]. Surprisingly, despite the considerable nutritional value (substantial content of dietary fiber and omega-3 fatty acids) and numerous health benefits of chia seed oil (e.g., therapeutic effects on the control of diabetes, dyslipidemia, and hypertension, as an anti-inflammatory, antioxidant, antidepressant, antianxiety, and analgesic) [164,169], the investigations of Nieman et al. [117] mentioned in presented review did not confirm its positive influence on sports performance. In turn, the lack of beneficial effects of the use of coconut oil noted by Borba et al. [114] seems to fit into the worldwide discussion about the health effects of its consumption. Despite many benefits, including weight loss, improvement of cognitive functions, irritable bowel syndrome, thyroid conditions, and diabetes, enhancing the immune system, and promoting wound healing, according to Neelakantan et al. [165,170], there is consistent and strong evidence showing that coconut oil has an adverse effect on the lipid parameters associated with cardio-metabolic human health. The aforementioned authors concluded that coconut oil consumption results in significantly higher LDL cholesterol than nontropical vegetable oils. Conversely, Newport and Dayrit [166,171], on the basis of an analysis of 26 studies on the effects of consumption of coconut oil conducted over the past forty years, claimed that the dietary recommendation to avoid consuming coconut oil due to its effects on lipid parameters is unjustified. At the same time, the performed survey documented the beneficial role of vegetable oils on sports injuries and gut function. The findings of Tang et al. [119], evidencing that the administration of dietary flaxseed oil enhanced the prognosis of acute anterior cruciate ligament rupture in sportspeople, correspond with the findings of Forelli et al. [167,172], who claimed that intake of camelina, rapeseed, and flax oils supports joint health. Moreover, the improvement of gut function after the intake of perilla oil observed in athletes by Kawamura et al. [116] is consistent with numerous animal experiments, and a cohort or clinical studies reviewed by Zou et al. [168,173] evidenced that edible plant oils can modulate the gut microbiota. At the same time, the beneficial effects of the external application of plant oils are worth mentioning.

The substantial use of olive oil, especially by athletes following recommendations of the Mediterranean diet, which increased after educational sessions, supports the findings of Gil-Caselles [169,174], who pointed out that the promotion of consumption of extra-virgin oil and the propagation of healthy dietary habits among sportspeople is still strongly desired. At the same time, it is worth adding that the Mediterranean diet is a viable dietary strategy for enhancing athletic performance, muscle strength, and overall health among professional and amateur athletes [175,176]. On the basis of the reviewed findings, the aforementioned authors pointed out the beneficial role of this dietary pattern among others on oxidative stress and inflammation, injury, and illness risk, as well as vascular and cognitive function. Nevertheless, due to the small amount of research in this area, as well as considerable heterogeneity in participant demographics, athletic level, and testing conditions across studies, many authors (e.g., [156,176,177]) simultaneously underlined the need for further research incorporating standardized dosing, homogeneous athlete populations, and controlled dietary conditions.

In turn, as documented in a limited number of investigations, the moderate or even low consumption of other oils (e.g., canola oil and flaxseed oil), despite the many advantageous effects of their key components [170,178], is intriguing and might be caused by various factors. One of these seems to be a trend in diet [171,172,173,174,179,180,181,182] and supplementation [183] focusing on the intake of carbohydrates and/or proteins. Furthermore, some sportspeople prefer the consumption of strongly recommended nuts and seeds (e.g., [178,179,184,185]), suggested as sources of healthy fats, particularly for vegetarian [178,179,186,187] and young [180,188] athletes. At the same time, it should be stated that the low consumption of coconut oil by sportspeople noted in the performed survey might be caused by the discrepancy among the marketing claims, responders’ own beliefs in its supposed health benefits, and scientific evidence. Additionally, some contraindications of vegetable oil intake should be mentioned here. Vegetable oils, particularly those rich in omega-6 fatty acids like linoleic acid, can be converted into arachidonic acid in the body, which promotes inflammation [8]. Moreover, repeatedly heating vegetable oils creates harmful compounds, which contribute to oxidative stress and can damage cells and tissues, impacting an athlete’s recovery and long-term health [189,190].

Although the performed review presents promising results, this study has some limitations. The first limitation might be caused by the arbitrary restriction introduced, limiting the literature search to the first 500 hits. A second limitation might be connected to the inclusion in this review solely of papers written in English. A further limitation might be connected to potential biases of the reviewed publications.

## 5. Conclusions

The performed investigations confirmed that the plants most frequently used in global edible oil production were also the most frequently mentioned in the descriptions of patented alimentary products for sportspeople. However, due to the fact that the beneficial effects of vegetable oils were evidenced solely in the case of a few plant species because of the absence of investigations, it might be concluded that further studies of the impact of vegetable oils on morphological, physiochemical, and biochemical indicators of sportspeople’s health are strongly needed. At the same time, future investigations should concentrate on the search for other promising plant oil sources. Considering the popularity of extra-virgin olive oil mainly in Mediterranean countries and the lower consumption of other vegetable oils that are advantageous for human health, it might be stated that the beneficial effects of plant oils with the optimal ratio for human health of ω-6 and ω-3 fatty acids still deserve widespread dissemination among amateur and professional athletes.

## Figures and Tables

**Table 1:** The names of families and plant species used as the oil source in patented inventions referring to sports nutrition published in the years 2015–2024.

Family	Taxon		Number of Patents
	Latin Name	Selected Common Name (s)	
*Anacardiaceae*	*Anacardium occidentale* L.	Cashew nut	1
*Apiaceae*	*Coriandrum sativum* L.	Coriander	1
*Arecaceae*	*Cyrtostachys renda* Blume	Sealing wax palm	2
	*Cocos nucifera* L	Coconut	29
*Asteraceae*	*Carthamus tinctorius* L.	Safflower	18
	*Helianthus annuus* L.	Sunflower	24
*Betulaceae*	*Corylus avellana* L.	Hazelnut	1
*Boraginaceae*	*Borago officinalis* L.	Borage	2
*Brassicaceae*	*Brassica juncea* (L.) Czern.	Brown mustard	1
	*Brassica napus* L.	Rapeseed; canola	26
	*Brassica oleracea* var. *italica* Plenck	Broccoli	1
	*Camelina sativa* (L.) Crantz	Camelina; false flax	1
	*Sinapis alba* L.	White mustard	1
*Cannabaceae*	*Cannabis sativa* L.	Hemp	3
*Cucurbitaceae*	*Citrullus lanatus* (Thunb.) Matsum. & Nakai	Bitter apple; bitter melon	1
	*Cucurbita pepo* L.	Pumpkin	1
*Elaeagnaceae*	*Hippophae rhamnoides* L.	Sea buckthorn	1
*Euphorbiaceae*	*Ricinus communis* L.	Castor oil plant	2
*Fabaceae*	*Arachis hypogaea* L.	Peanut; groundnut	22
	*Glycine max* (L.) Merr.	Soybean; soya	32
*Fagaceae*	*Fagus sylvatica* L	Common beech	1
*Grossulariaceae*	*Ribes nigrum* L.	Black currant; blackcurrant	3
*Juglandaceae*	*Carya illinoinensis* (Wangenh.) K.Koch	Pecan	1
	*Juglans major* (Torr.) A. Heller	Arizona walnut	1
	*Juglans regia* L.	Common walnut	4
*Lamiaceae*	*Salvia hispanica* L.	Chia	1
*Lauraceae*	*Persea americana* Mill.	Avocado	4
*Lecythidaceae*	*Bertholletia excelsa* Humb. & Bonpl.	Brazil nut	1
*Linaceae*	*Linum usitatissimum* L.	Flax; flaxseed; common flax	13
*Malvaceae*	*Gossypium arboreum* L.	Cotton; tree cotton	11
*Oleaceae*	*Olea europaea* L.	Olive	33
*Onagraceae*	*Oenothera biennis* L.	Evening primrose	5
*Pedaliaceae*	*Sesamum indicum* L.	Sesame	14
*Poaceae*	*Oryza sativa* L.	Rice	4
	*Triticum aestivum* L.	Common wheat	1
	*Zea mays* L.	Maize; corn	21
*Ranunculaceae*	*Nigella sativa* L.	Black cumin	1
*Rosaceae*	*Prunus amygdalus* Batsch	Almond	4
*Vitaceae*	*Vitis vinifera* L.	Common grape	4

**Table 2:** A review of original articles devoted to the effects of plant oils on the health and athletic performance of sportspeople in alphabetical order by the lead author of the studies.

Author(s) and Year of Publication	Country	Sports Discipline Sex; Age (Mean ± SD or Years); Number of Participants	Treatment	Duration	Activity
Ayari and Boukazoula (2023) [113]	Not specified	Half-marathon M; 19–22; 30	Group 1: untrained runners receiving 20 mL/day of virgin olive oil (control); Group 2: half-marathon runners performing training routines 5 days a week, receiving 20 mL/day of olive oil; Group 3: half-marathon runners performing training routines 5 days a week and unsupplemented with virgin olive oil.	10 weeks	Changes among test groups Similar levels of T, LH, FSH, CORT, and insulin at baseline; The greatest level of T and LH in Group 2, as well as CORT in Group 3, before and immediately after a marathon race, as well as 24 h after a race. Changes from baseline ↔ Level of insulin and FSH in all groups; ↑ T level in Group 2 and ↑ CORT in Group 3.
Borba et al. (2019) [114]	Brazil	Recreational run -; 28.46 ± 5.63; 13	Consumption of (1) Placebo water (control); (2) Decaffeinated coffee plus isolated caffeine (test group); (3) Decaffeinated coffee plus isolated caffeine plus soy oil (test group); (4) Decaffeinated coffee plus isolated caffeine plus extra-virgin coconut oil (test group). The substances were ingested 60 min before a 1600 m time trial at a 400 m track.	4 sessions separated by one-week intervals	After supplementation In all groups, ↔ RPE and running time. Changes from baseline In all groups, ↔ Blood lactate concentration.
Capó et al. (2016) [106]	Spain	Taekwondo M; 22.8 ± 3.8; 5 (young sportspeople) Athletics M; 45.6 ± 1.6; 5 (senior sportspeople)	Consumption of 1 L of almond beverage containing olive oil five days a week. Half of the beverage was taken in the morning, and the other half before the daily training session. The results obtained after the nutritional intervention (supplemented groups) were compared with those obtained at the beginning of the intervention (control groups).	5 weeks	After supplementation ↑ TNFα and ↑ 15LOX2 gene expression and IL1β; ↔ IL10, IL15, HSP72, NFκβ, and TLR4. After exercise ↑ DHA in erythrocytes; ↑ TNFα gene expression in PBMCs in young sportspeople; ↑ 15LOX2 gene expression, especially in the senior group; ↔ Expression of TLR4, NFκβ, 5LOX, IL-10, IL-15, and HSP72 in PBMCs; ↑ Plasma NFEAs, sICAM3, and sL-selectin; ↓ LX in the senior group.
Capó et al. (2016b) [107]	Spain	Taekwondo M; 22.8 ± 3.8; 5 (young sportspeople) Athletics M; 45.6 ± 1.6; 5 (senior sportspeople)	Consumption of 1 L of almond beverage containing olive oil five days a week. Half of the beverage was taken in the morning, and the other half before the daily training session. The results obtained after the nutritional intervention (supplemented groups) were compared with those obtained at the beginning of the intervention (control groups).	5 weeks	After supplementation ↑ PUFA, ↑ DHA erythrocyte content, and ↓ SFA; ↔ blood polyphenol levels; ↑ RBC concentration in senior athletes; ↓ Plasma MDA concentration; ↑ Hemoglobin (Hb) (g per 100 mL) in the senior group. After exercise ↔ HCT (%), NOx plasma levels, nitrite (nM), LPO, CI, SFA, and PUFA in all groups; ↑ Hb in all groups; ↓ Blood polyphenols and RBCs in all groups; ↑ CI in the senior groups; ↑ MUFA in the young groups.
Esquius et al. (2019) [108]	Spain	Running M; 22.2 ± 4.3; 7	Dietary supplementations in a randomized order: (i) 25 mL of extra-virgin olive oil, (ii) 25 mL of palm oil, (iii) and 8 g of a placebo.	Three effort sessions separated by 7-day intervals	Changes from baseline ↑ Ventilation efficiency and cardiorespiratory system in olive oil supplementation compared with palm oil; ↔ Moderate exercise intensity with palm oil and placebo supplementation; ↔ High exercise intensity with supplementation; ↔ Exercise time with supplementation.
Esquius et al. (2021) [109]	Spain	Recreational sports training M; 35–51; 3	Dietary supplementations: (i) active supplement (100 mL of commercial orange juice, 8 g of modified starch, and 25 mL of extra-virgin olive oil); (ii) placebo (100 mL of commercial orange juice and 8 g of modified starch).	Twice, separated by 7-day interval	Changes from baseline ↓ DC (markers of inflammatory process) after active supplement intake.
Kamoun et al. (2021) [115]	Tunisia	Recreational strength and endurance training M; 66.5 ± 2.68; 10 (test group) M; 66.9 ± 2.13; 10 (control)	Test group: concurrent training + dietary walnut consumption (15 g/day). Control: concurrent training + control diet.	6 weeks	Test group vs. control group In the test group, ↓ TC, LDL, and TG; ↓ CRP; ↑ HDL.
Kawamura et al. (2023) [116]	Japan	Volleyball F; 20.2 ± 1.3; 36 12 athletes (test group 1) 12 athletes (test group 2) 12 athletes (control)	Test group 1: 9 g/day of perilla oil. Test group 2: 3 g/day of perilla oil. Control: placebo.	8 week	Test group vs. control group In test group 1, ↓ Urinary IS; ↓ Spoilage bacteria (Proteobacteria); ↑ Butyrate-producing bacteria (Lachnospiraceae).
Mielgo-Ayuso et al. (2020) [110]	Spain	Marathon race M; 44.94 ± 8.77; 69	2–4 servings of olive oil per day, 7 days before the race.	One week	Changes from baseline ↓ TNI and TNT post-marathon concentration as markers of EIMD and EICS, respectively.
Mieszkowski et al. (2020) [111]	Poland	Ultramarathon race M; 42.00 ± 8.44; 13 (test group) M; 40.00 ± 8.11; 14 (control)	Test group: a single dose (150,000 IU) of vitamin D, as a solution in 10 mL of vegetable oil, 24 h before starting the race. Control: placebo.	One day	Test group vs. control group ↑ Serum 25(OH)D3, 24,25(OH)2D3, and 3-epi-25(OH)D3 levels significantly after supplementation. Changes from baseline ↑ Serum 25(OH)D3, 24,25(OH)2D3, and 3-epi-25(OH)D3 levels significantly after the ultramarathon in test and control groups.
Mieszkowski et al. (2021) [112]	Poland	Ultramarathon race M; 42.40 ± 7.59; 16 (test group) M; 39.48 ± 6.89; 19 (control)	Test group: a single dose (150,000 IU) of vitamin D, as a solution in 10 mL of vegetable oil, 24 h before starting the race. Control: placebo.	One day	Test group vs. control group ↑ Serum 25(OH)D in the test group. Changes from baseline ↑ IL 6 and 10 IL-6; ↑ IL10 and resistin levels immediately after the run, especially in the control group; ↓ Leptin, ↓ OSM, and ↓ TIMP levels.
Nieman et al. (2015) [117]	USA	Running M; 24–55; 16 F; 24–55; 8	Test group: 0.5 L of water with chia seed oil (0.43 g of ALA/BM) + run. Control group: 0.5 L of water + run.	Twice separated by two weeks	Test group vs. control group In the test group, post-run, ↑ Plasma ALA; ↔ RER and run time to exhaustion, ↔ oxygen consumption, ↔ ventilation, and ↔ blood lactate. Changes from baseline ↑ Leukocyte number, ↑ plasma ALA, ↑ cortisol (CORT), and ↑ IL-6; ↑ IL8, ↑ IL10, and ↑ TNF-α.
Sinaga (2017) [118]	Not specified	Athletics -; 20–23; 15 (test group) -; 20–23; 15 (control group)	Test group: consumption of red fruit oils once a day after meal.	3 months	Test group vs. control group ↑ VO_2_max, ↑ number of erythrocytes, and ↑ levels of Hb and HCT in the test group.
Tang et al. (2022) [119]	China	Ball sports, running, climbing, dancing, swimming, and fitness M; 15–44; 50 (test group) F; 15–44; 21 (test group) M; 15–45; 47 (control) F; 15–45; 24 (control)	Test group: 6 flaxseed oil capsules daily. Control: 6 corn oil capsules daily.	2 years	Test group vs. control group ↑ IKDC and KOOS scores after two-year administration in test group.

Explanations of symbols and abbreviations: ↑—increase; ↓—decrease; ↔—no changes; ALA—alpha-linolenic acid; CI—carbonyl index; CORT—cortisol; CRP—C-reactive protein; DC—dendritic cell; DHA—docosahexaenoic acid; EICS—exercise-induced cardiac stress; EIMD—exercise-induced muscle damage; FSH—follicle-stimulating hormone; Hb—hemoglobin; HCT—hematocrit; HDL—high-density lipoprotein; HSP72—heat shock protein 72; IKDC– International Knee Documentation Committee; IL1β—interleukin-1 beta; IL 6—interleukin 6; IL8—interleukin 8; IL10—interleukin 10; IL15—interleukin 15; IS—indoxyl sulphate; KOOS—knee injury and osteoarthritis outcome; LDL– low-density lipoprotein; LH—luteinizing hormone; LPO—lipoperoxide; LX—lipoxin; NFEAs—non-esterified fatty acids; MDA—oxidative damage marker; NFκβ—nuclear factor kappa B; NOx—nitrogen oxide; OSM—oncostatin M; PBMCs—peripheral blood mononuclear cells; RBC—blood cell polyphenol; sICAM-3—soluble intercellular adhesion molecule 3; T—testosterone; TC—total cholesterol; TG—triglyceride; TIMP—tissue inhibitor of metalloproteinase; TLR4—toll-like receptor 4; TNFα—tumor necrosis factor α; TNI—cardiac troponin I; TNT—cardiac troponin T; sICAM3—soluble intercellular adhesion molecule 3; sL-selectin—soluble L-selectin; VO_2_max—volume of O_2_ maximum; 5LOX—5-lipoxygenase; 15LOX2—15-lipoxygenase-2; 25(OH)D3—3-epi-25-hydroxyvitamin D_3_; 24,25(OH)2D_3_—24,25-dihydroxyvitamin D_3_; and 25(OH)D_3_—25-hydroxyvitamin D_3_.

**Table 3:** The risk of bias in the following domains: D1—bias arising from the randomization process; D2—bias due to deviations from intended interventions; D3—bias due to missing outcome data; D4—bias in measurement of the outcome; and D5—bias in selection of the reported result of findings included into the narrative review. Symbols: L—low bias risk; SC—some concern; and ?—lack of information.

Author(s) and Year of Publication	Risk of Bias Domains					
	D1	D2	D3	D4	D5	Overall
Ayari and Boukazoula (2023) [113]	?	L	L	L	L	SC
Borba et al. (2019) [114]	L	L	L	L	L	L
Capó et al. (2016) [106]	?	L	L	L	L	SC
Capó et al. (2016b) [107]	?	L	L	L	L	SC
Esquius et al. (2019) [108]	L	L	L	L	L	L
Esquius et al. (2021) [109]	L	L	?	L	L	SC
Kamoun et al. (2021) [115]	L	L	L	L	L	L
Kawamura et al. (2023) [116]	L	L	L	L	L	L
Mielgo-Ayuso et al. (2020) [110]	?	L	?	L	L	SC
Mieszkowski et al. (2020) [111]	L	L	L	L	L	L
Mieszkowski et al. (2021) [112]	L	L	L	L	L	L
Nieman et al. (2015) [117]	L	L	L	L	L	L
Sinaga (2017) [118]	?	L	L	?	L	SC
Tang et al. (2022) [119]	L	L	L	L	L	L

**Table 4:** A review of questionnaire surveys on the frequency of use of plant oils by sportspeople in alphabetical order by the lead author of the studies. Abbreviations: F—females, M—males, –—lack of data.

Author(s) and Year of Publication	Country	Sports Discipline	Sex; Age (Mean ± SD or Range of Years); Number of Participants	Plant Oil	Use of Plant Oils
Amawi et al. (2023) [127]	Saudi Arabia	Soccer	M; 19 ±1; 81	Olive oil	40 (49.4%) respondents declared consumption of olive oil
Alahmadi, Albassam (2023) [128]	Saudi Arabia	Athletics	M,F; 26.41 ± 8.1; 261	Olive oil	46.1% of respondents confirmed use of olive oil for cooking or baking
Gacek and Frączek (2016) [129]	Poland	Football	M; 17–19; 303	Olive oil and other plant oils	Olive oil is consumed less frequently than other plant oils
Hooks et al. (2023) [136]	Ireland	Hockey; cricket	F; 24.8 ± 4.5; 35	Canola oil; flaxseed oil	20 athletes consume canola oil, while 2 respondents consume flaxseed oil
Kontele et al. (2021) [121]	Greece	Gymnastics	F; 11–18; 319	Olive oil	93.3% of participants reported using olive oil at home
Leão et al. (2023) [123]	Portugal	Soccer	M; 12.0 ± 2.2; 132	Olive oil	131 (99%) of respondents declared the use of olive oil at home
Martínez–Rodríguez et al. (2021) [133]	Spain	Handball	M; 17.0 ± 0.1; 14 (junior) M; 25.5 ± 4.7; 24 (senior) F; 16.1 ± 1.46; 7 (junior) F; 23.2 ± 2.9; 14 (senior)	Olive oil	The use of olive oil at home was confirmed by 7 (33%) junior females, 14 (68%) senior females, 13 (34%) junior males, and 23 (60%) senior males
Martinovic et al. (2021) [126]	Croatia	Fitness	F; 30.3 ± 9.9; 530 M; 28.2 ± 7.8; 690	Olive oil	The use of olive oil was confirmed by 34% of the participants
Martinovic et al. (2022) [134]	Croatia	Athletics	M; 24.5 ± 4.0; 87 (professional athletes) F; –; 63 (professional athletes) M; 24.0 ± 5.5; 78 (recreational athletes) F; –; 72	Olive oil	The use of olive oils was confirmed by 52 (34.7%) professional athletes and 21 (14.0%) recreational athletes
Muros and Zabala (2018) [120]	Spain	Cycling; triathlon	F,M; 34.14 ± 9.28; 4037	Olive oil	95% of the participants use olive oil as the principal source of fat for cooking 4 or more tablespoons of olive oil per day is consumed by 78.5% of the participants from southern Spain and 74.1% of the participants from northern Spain
Muros et al. (2021) [132]	Spain	Cycling; triathlon	F,M; 34.14 ± 9.28; 4037	Olive oil	The number of servings of olive oil per day reaches 1.36 ± 1.16 among males, 1.88 ± 1.62 among females, 1.47 ± 1.25 among triathletes, and 1.35 ± 1.20 among cyclists
Novokshanova (2021) [137]	Russia	Athletics	F,M; –; 1267	Vegetable oils	215 (17%) of the respondents use vegetable oils as a source of fat
Peláez–Barrios, Vernetta (2022) [135]	Spain	Acrobatic gymnastics	F; 13.69 ± 3.05; 81 (gymnasts) F; 14.04 ± 1.49; 70 (non-gymnasts)	Olive oil	The use of olive oil was reported by 80 (98.8%) gymnasts and 51 (72.9%) non-gymnasts
Philippou et al. (2017) [125]	Cyprus	Swimming	F; –; 11 M; –; 23	Olive oil	71% of respondents used olive oil before the nutrition education workshop 82% of respondents used olive oil after the nutrition education workshop
Ritz et al. (2020) [138]	USA	Baseball, basketball, cross country, football, golf, gymnastics, soccer, softball, swimming and diving, track and field, and volleyball	F,M; >18 years; 1562	Canola oil	Canola oil is consumed by 85% of the participants, while flax oil or flax is consumed by 34.9% of the participants
Santana et al. (2019) [131]	Spain	Rhythmic gymnastics	F; 7–12; 124 (younger) F; 13–17; 97 (adolescents)	Olive oil	94.4% of younger and 88.7% of adolescent gymnasts declared use of olive oil
Santos-Sánchez et al. (2021) [122]	Spain	Soccer	M; 8–12; 75	Olive oil	100% of respondents declared the use of olive oil at home
Staśkiewicz e al. (2022) [139]	Poland	Bodybuilding, CrossFit, football, and handball	M; 23.90 ± 4.08; 30 (professional football players) M; 24.37 ± 4.15; 30 (professional handball players) M; 25.00 ± 4.00; 33 (amateur bodybuilding sportspeople) M; 24.30 ± 3.78; 26 (amateur CrossFit sportspeople)	Vegetable oils	Frequency of participants who used 1 tablespoon of oils reached 3.70 ± 1.44 in the case of the amateur players and 3.33 ± 1.17 in the case of the professional players Frequency of participants who used 1 tablespoon of oils ranged from 3.30 ± 0.54 (handball players) to 3.37 ± 0.46 (football players), 3.54 ± 0.37 (CrossFit), and 3.54 ± 0.37 (bodybuilders)
Szot et al. (2023) [130]	Poland	Esport	M; 20.5 ± 2.0; 233	Canola oil; coconut oil; olive oil	43.35% of the respondents consume olive oil and canola oil 1–3 times per month, 18.03% of the respondents consume them once a day, and 0.43% consume them 4–5 times a day; the mean frequency of consumption of coconut oil reaches 2.17 ± 1.42 servings
Ventura Comes et al. (2018) [140]	Spain	Squash	M; –; 10 (international players) F; –; 4 (international players) M; –; 20 (national players) F; –; 8 (national players)	Coconut oil; flaxseed oil	Flaxseed oil is consumed by 4 (28.6%) international players and 1 (3.6%) national player. Coconut oil is consumed by 3 (21.4%) international players and 2 (7.1%) national players
Vélez-Alcázar et al. (2024) [124]	Spain	Athletics	M; 18.31 ± 2.31; 47 F; 17.27 ± 1.44; 49	Olive oil	The use of olive oil at home is declared by 57 (96.6%) of respondents with excellent, 29 (96.7%) with moderate, and 5 (71.4%) with poor adherence to the Mediterranean diet

## Data Availability

All data are presented in this article.

## Appendix A

**Table A1:** The characteristics of patents referring to sports nutrition containing plant-derived oils, according to the alphabetical order of the main inventor.

	Main Inventor Name	Patent Title	Plant Species	Activity	Consistency (Form) of Invention	Reference
1.	Amyx, L.	Sports and nutritional supplement formulations	*Brassica napus* L. *Cocos nucifera* L. *Glycine max* (L.) Merr. *Helianthus annuus* L. *Zea mays* L.	Muscle strength improvement; sports performance improvement	Liquid (beverage and suspension), solid (capsules, tablets, and lozenges), semi-solid (gel), and bulk-solid (powder)	[48]
2.	Andreeva, L.	Nutritional compositions for skeletal muscle	*Arachis hypogaea* L. *Glycine max* (L.) Merr. *Gossypium arboreum* L. *Olea europaea* L *Zea mays* L.	Muscle strength improvement	Liquid (beverage)	[49]
3.	Asada, M.	Sports drink	*Arachis hypogaea* L. *Brassica napus* L. *Carthamus tinctorius* L. *Cocos nucifera* L. *Glycine max* (L.) Merr. *Helianthus annuus* L. *Olea europaea* L *Oryza sativa* L. *Zea mays* L.	Nutrition	Liquid (beverage)	[50]
4.	Barata, M.	Nutritional formulations comprising a pea protein isolate	*Brassica napus* L. *Carthamus tinctorius* L. *Cocos nucifera* L. Glycine max (L.) Merr. *Gossypium arboreum* L. *Helianthus annuus* L. *Olea europaea* L *Vitis vinifera* L. *Zea mays* L.	Nutrition and weight loss	Solid (bar, cakes, and cookies), bulk-solid (powder), and liquid (beverage)	[51]
5.	Bartos, J.D.	Endurance formulation and use	*Olea europaea* L.	Endurance improvement	Liquid (beverage, syrup, and emulsion), solid (capsules, tablets, lozenges, chewing gum, and bars), semi-solid (gel), and bulk-solid (powder)	[52]
6.	Bermond, G.	Compositions for the improvement of sports performance	*Arachis hypogaea* L. *Cocos nucifera* L. *Olea europaea* L. *Sesamum indicum* L.	Sports performance improvement	Solid (capsules, tablets, and lozenges), bulk-solid (powder and granules), and liquid (emulsion and syrup)	[53]
7.	Billecke, N.	Nutritional composition and process for preparing it	*Arachis hypogaea* L. *Bertholletia excelsa* Humb. & Bonpl. *Brassica napus* L. *Carthamus tinctorius* L. *Cocos nucifera* L. *Coriandrum sativum* L. *Corylus avellana* L. *Glycine max* (L.) Merr. *Helianthus annuus* L. *Juglans major* (Torr.) A. Heller *Linum usitatissimum* L. *Olea europaea* L *Persea americana* Mill. *Ricinus communis* L. *Sesamum indicum* L. *Sinapis alba* L. *Zea mays* L.	Nutrition	Liquid (beverage) and bulk-solid (powder)	[54]
8.	Bortz, J.D.	Composition for improved performance	*Arachis hypogaea* L. *Brassica napus* L. *Cannabis sativa* L. *Carthamus tinctorius* L. *Cocos nucifera* L. *Glycine max* (L.) Merr. *Linum usitatissimum* L. *Olea europaea* L. *Triticum aestivum* L. *Zea mays* L.	Sports performance improvement	Liquid (beverage), solid (tablets and capsules), semi-solid (gel), and bulk-solid (powder)	[55]
9.	Bos, P.	Novel combinations comprising konjac mannan, and their compositions and uses	*Borago officinalis* L. *Cocos nucifera* L. *Linum usitatissimum* L. *Oenothera biennis* L. *Olea europaea* L. *Ribes nigrum* L.	Weight loss	Bulk-solid (powder and granules)	[56]
10.	Burke, L.M.	Method of enhancing muscle protein synthesis	*Brassica napus* L. *Helianthus annuus* L. *Zea mays* L.	Muscle mass increase	Liquid (beverage), semi-solid (gel), and bulk-solid (powder)	[57]
11.	Do, P.P.	Composition and method for nutrition formulation	*Anacardium occidentale* L. *Arachis hypogaea* L. *Brassica napus* L. *Carthamus tinctorius* L. *Carya illinoinensis* (Wangenh.) K.Koch *Citrullus lanatus* (Thunb.) Matsum. & Nakai *Cocos nucifera* L. *Fagus sylvatica* L. *Gossypium arboreum* L. *Helianthus annuus* L. *Juglans regia* L. *Olea europaea* L. *Persea americana* Mill. *Pistacia vera* L. *Prunus amygdalus* Batsch *Sesamum indicum* L. *Vitis vinifera* L	Sport performance improvement and post-exercise recovery support	Liquid (beverage) and solid (bar)	[58]
12.	Du, Y.	Instant soybean protein composition and preparation method	*Cocos nucifera* L. *Helianthus annuus* L. *Linum usitatissimum* L.	Nutrition	Bulk-solid (powder)	[59]
13.	Dyrvig, M.	Neutral instant beverage whey protein powder	*Brassica napus* L. *Glycine max* (L.) Merr.	Nutrition	Bulk-solid (powder)	[60]
14.	Feng, W.	Compositions for post-exercise recovery and methods of making and using	*Arachis hypogaea* L. *Brassica napus* L. *Carthamus tinctorius* L. *Cocos nucifera* L. *Glycine max* (L.) Merr. *Helianthus annuus* L. *Juglans regia* L. *Linum usitatissimum* L. *Olea europaea* L. *Prunus amygdalus* Batsch *Salvia hispanica* L. *Sesamum indicum* L. *Vitis vinifera* L. *Zea mays* L.	Post-exercise recovery support	Solid (chewing gum) and semi-solid (gel)	[61]
15.	Francis, C.	Methods and compositions comprising caffeine and/or a derivative and a polyphenol	*Brassica napus* L. *Cocos nucifera* L. *Glycine max* (L.) Merr. *Gossypium arboreum* L. *Olea europaea* L. *Prunus amygdalus* Batsch *Ribes nigrum* L. *Sesamum indicum* L. *Zea mays* L.	Cognitive function improvement	Liquid (beverage), solid (chewing gum, tablets, and capsules), semi-solid (gel), and bulk-solid (powder and granules)	[62]
16.	Franse, M.M.	Protein bar	*Arachis hypogaea* L. *Brassica napus* L. *Camelina sativa* (L.) Crantz *Carthamus tinctorius* L. *Glycine max* (L.) Merr. *Helianthus annuus* L. *Olea europaea* L. *Oryza sativa* L. *Sesamum indicum* L. *Zea mays* L.	Nutrition	Solid (bar)	[63]
17.	George, M.	Healthful supplements	*Cannabis sativa* L. *Cocos nucifera* L.	Health improvement	Liquid (beverage) and solid (capsules and tablets)	[64]
18.	He, X.	A kind of plant energy rod suitable for sportspeople and preparation method	*Helianthus annuus* L.	Fatigue delay	Solid (bar)	[65]
19.	Hwang, J.-G.	Composition for increase in muscular functions or exercise capacity comprising Vigna unguiculata (L.) Walp., Vicia faba L., or Dolichos lablab L.	*Olea europaea* L.	Sport performance improvement	Liquid (suspension and syrup), solid (capsules and tablets), and bulk-solid (granules and powder)	[66]
20.	Karaboga, A.S.	Sublingual compositions comprising natural extracts and uses	*Arachis hypogaea* L. *Brassica napus* L. *Glycine max* (L.) Merr.	Joint and muscular pain relief and endurance improvement	Solid (capsules, tablets, and pills), semi-solid (gel, cream, paste, and ointments), and bulk-solid (powder and granules)	[67]
21.	Kawamura, H.	Chewable tablet	*Arachis hypogaea* L. *Brassica napus* L. *Carthamus tinctorius* L. *Cocos nucifera* L. *Glycine max* (L.) Merr. *Oenothera biennis* L. *Oryza sativa* L. *Persea americana* Mill. *Sesamum indicum* L.	Post-exercise recovery support	Solid (tablets)	[68]
22.	Kim, S.-J.	Composition for muscle strengthening, physical performance improvement, and muscle fatigue recovery	*Olea europaea* L.	Post-exercise recovery support and sport performance improvement	Solid (capsules, pills, tablets, and chewing gum), semi-solid (cream and gel), and bulk-solid (granules)	[69]
23.	Kleidon, W.	Methods and compositions for enhancing health	*Arachis hypogaea* L. *Carthamus tinctorius* L. *Cocos nucifera* L. *Glycine max* (L.) Merr. *Gossypium arboreum* L. *Sesamum indicum* L. *Zea mays* L.	Health improvement	Liquid (beverage, suspension, and emulsion), semi-liquid (slurry), solid (tablets and capsules), semi-solid (gel), and bulk-solid (powder and granules)	[70]
24.	Komorowski, J.R.	Chromium-containing compositions for improving health and fitness	*Arachis hypogaea* L. *Cocos nucifera* L. *Olea europaea* L. *Sesamum indicum* L.	Muscle mass increase and muscle strength improvement	Liquid (beverage, elixir, emulsion, and syrup), solid (capsules and tablets), and bulk-solid (powder and granules)	[71]
25.	Konopacki, A.	Prepared foods having high-efficacy omega-6/omega-3-balanced polyunsaturated fatty acids	*Arachis hypogaea* L. *Brassica napus* L. *Glycine max* (L.) Merr. *Helianthus annuus* L. *Linum usitatissimum* L. *Vitis vinifera* L. *Zea mays* L.	Nutrition	Liquid (beverage)	[72]
26.	Lauridsen, K.B.	Instant beverage powder based on blg	*Brassica napus* L. *Cocos nucifera* L. *Helianthus annuus* L.	Nutrition	Bulk-solid (powder)	[73]
27.	Laursen, R.R.	Nutraceutical composition for mental activity	*Helianthus annuus* L.	Mental activity improvement	Semi-solid (gel), bulk-solid (powder), and solid (capsules, pills, cookies, and bars)	[74]
28.	Liu, F.	Protein composition for rapid energy replenishment	*Helianthus annuus* L.	Nutrition	Solid (bar)	[75]
29.	Longo, V.	A diet composition for enhancing lean body mass and muscle mass	*Brassica napus* L. *Cocos nucifera* L. *Olea europaea* L.	Muscle mass increase	Liquid (soup)	[76]
30.	Maevsky, E.I.	Formulations and dosage forms for enhancing performance or recovery from stress	*Cocos nucifera* L. *Glycine max* (L.) Merr. *Gossypium arboreum* L. *Helianthus annuus* L.	Sports performance improvement and post-exercise recovery support	Liquid (syrup and emulsion), solid (capsules, tablets, and lozenges), and bulk-solid (powder)	[77]
31.	Mann, S.J.	Composition comprising a source of nitrate derived from amaranthus leaf and/or rhubarb	*Carthamus tinctorius* L. *Glycine max* (L.) Merr. *Helianthus annuus* L. *Oenothera biennis* L. *Olea europaea* L.	Muscle strength improvement	Solid (capsules, tablets, and lozenges), semi-solid (gel), and bulk-solid (powder and granules)	[78]
32.	Martinez, A.	Methods of reducing exercise-induced injury or enhancing muscle recovery after exercise	*Brassica napus* L. *Carthamus tinctorius* L. *Glycine max* (L.) Merr.	Post-exercise recovery support	Liquid (emulsion, suspension, syrup, and elixir), solid (capsules, tablets, chewing gum, pills, and lozenges), and bulk-solid (granules and powder)	[79]
33.	Mathisen, J.S.	Omega-3 beverage	*Nigella sativa* L.	Nutrition	Liquid (beverage)	[80]
34.	Miller, P.J.	Compositions and methods for increasing mitochondrial activity	*Arachis hypogaea* L. *Borago officinalis* L. *Brassica juncea* (L.) Czern. *Brassica napus* L. *Carthamus tinctorius* L. *Cocos nucifera* L. *Cucurbita pepo* L. *Glycine max* (L.) Merr. *Gossypium arboreum* L. *Helianthus annuus* L. *Juglans regia* L. *Linum usitatissimum* L. *Oenothera biennis* L. *Olea europaea* L. *Persea americana* Mill. *Prunus amygdalus* Batsch *Ribes nigrum* L. *Sesamum indicum* L.	Sports performance improvement	Solid (capsules) and bulk-solid (powder)	[81]
35.	Miura, K.	Composition for improving muscular endurance	*Glycine max* (L.) Merr. *Olea europaea* L. *Ricinus communis* L. *Sesamum indicum* L.	Endurance improvement	Liquid (suspension), solid (capsules and tablets), semi-solid (gel and cream), and bulk-solid (powder and granules)	[82]
36.	Morita, H.	Composition for improving athletic performance	*Glycine max* (L.) Merr. *Olea europaea* L. *Zea mays* L.	Sports performance improvement	Liquid (suspension and syrup), solid (tablets and capsules), and bulk-solid (powder and granules)	[83]
37.	Morris, S.R.	Formulation for increasing energy	*Arachis hypogaea* L. *Brassica oleracea* var. *italica* Plenck *Cocos nucifera* L. *Olea europaea* L. *Sesamum indicum* L.	Cognitive function improvement and endurance improvement	Liquid (suspension and syrup), semi-liquid (slurry), solid (tablets, pills, and capsules), and semi-solid (gel)	[84]
38.	Nielsen, S.B.	pH-neutral beta-lactoglobulin beverage preparation	*Brassica napus* L.	Nutrition	Liquid (beverage)	[85]
39.	Pang, F.	Sports fermented milk and preparation method	*Arachis hypogaea* L. *Brassica napus* L. *Glycine max* (L.) Merr. *Helianthus annuus* L. *Linum usitatissimum* L. *Zea mays* L.	Post-exercise recovery support and nutrition	Liquid (beverage)	[86]
40.	Phillips, S.	Multi-nutrient composition	*Arachis hypogaea* L. *Brassica napus* L *Carthamus tinctorius* L. *Cocos nucifera* L. *Glycine max* (L.) Merr. *Helianthus annuus* L. *Juglans regia* L. *Linum usitatissimum* L. *Olea europaea* L. *Zea mays* L.	Muscle mass increase, muscle strength improvement, and cognitive function improvement	Liquid (beverage, suspension, and emulsion), semi-solid (gel), solid (bar, tablets, and capsules), and bulk-solid (powder)	[87]
41.	Pradera Bañuelos, D.	Biscuit bar with a moderate sugar content, made with whole grains, nuts, seeds, and extra-virgin olive oil	*Olea europaea* L.	Nutrition	Solid (bar)	[88]
42.	Rischbieter, I.	High-energy food supplement based on inverted sugars and ergogenic products for use in physical activity and method for producing it	*Arachis hypogaea* L. *Brassica napus* L. *Cocos nucifera* L. *Cyrtostachys renda* Blume *Glycine max* (L.) Merr. *Helianthus annuus* L. *Olea europaea* L. *Zea mays* L.	Sports performance improvement	Liquid (beverage) and semi-solid (gel and paste)	[89]
43.	Ryutaro, Y.	Liquid nutritional composition	*Arachis hypogaea* L. *Brassica napus* L. *Carthamus tinctorius* L. *Cocos nucifera* L. *Glycine max* (L.) Merr. *Helianthus annuus* L. *Olea europaea* L. *Oryza sativa* L. *Zea mays* L.	Post-exercise recovery support and nutrition	Bulk-solid (powder)	[90]
44.	Scheiman, J.	Compositions and methods for enhancing exercise endurance	*Arachis hypogaea* L. *Carthamus tinctorius* L. *Glycine max* (L.) Merr. *Gossypium arboreum* L. *Olea europaea* L. *Sesamum indicum* L. *Zea mays* L.	Endurance improvement	Solid (pills, tablets, and capsules)	[91]
45.	Sheng, G.	Sports supplement suitable for long-term bodybuilding and preparation method	*Carthamus tinctorius* L. *Cyrtostachys renda* Blume *Cocos nucifera* L. *Glycine max* (L.) Merr. *Olea europaea* L.	Muscle mass increase	Bulk-solid (powder)	[92]
46.	Shi, K.	Omega-3 fatty acid-enriched lipidosome exercise drink and preparation method	*Linum usitatissimum* L.	Post-exercise recovery support	Liquid (beverage)	[93]
47.	Skladtchikova, G.N.	Nutritional compositions	*Arachis hypogaea* L. *Glycine max* (L.) Merr. *Gossypium arboreum* L. *Olea europaea* L. *Zea mays* L.	Nutrition	Liquid (beverage, suspension, and syrup) and solid (tablets, capsules, wafers, and chewing gums)	[94]
48.	Steinfeld, U.	Dietary supplements, uses, methods of supplementation, and oral spray	*Cocos nucifera* L.	Mental and physical stress reduction	Liquid (aqueous solution and emulsion)	[95]
49.	Uberti, F.	Vegetable oil composition and uses	*Cannabis sativa* L. *Linum usitatissimum* L.	Health improvement	Solid (tablets, lozenges, and pills) and bulk-solid (granules)	[96]
50.	Van Riet, N.	Food supplement for improving sports performance	*Olea europaea* L.	Sports performance improvement	Liquid (solution, suspension, and emulsion), solid (capsules and tablets), bulk-solid (powder), and semi-solid (gel)	[97]
51.	Xie, Y.	Composition for promoting health of sportspeople and preparation method	*Arachis hypogaea* L. *Glycine max* (L.) Merr. *Hippophae rhamnoides* L. *Sesamum indicum* L.	Health improvement	Bulk-solid (powder)	[98]
52.	Xu, Q.	High-protein dietary fiber energy protein bar and production method	*Cocos nucifera* L. *Helianthus annuus* L.	Post-exercise recovery support	Solid (bar)	[99]
53.	Xu, S.	Double-protein sports milk and preparation method	*Brassica napus* L.	Post-exercise recovery support, nutrition, and sports performance improvement	Liquid (beverage)	[100]
54.	Yang, J.	A kind of peracidity sports nutrition liquid and its preparation process	*Brassica napus* L. *Carthamus tinctorius* L. *Cocos nucifera* L. *Glycine max* (L.) Merr. *Gossypium arboreum* L. *Helianthus annuus* L. *Linum usitatissimum* L. *Oenothera biennis* L. *Olea europaea* L. *Zea mays* L.	Post-exercise recovery support, muscle strength improvement, and endurance improvement	Liquid (beverage)	[101]
55.	Zhao, Y.	Energy gel with antioxidation function and preparation method	*Olea europaea* L.	Nutrition	Semi-solid (gel)	[102]
56.	Zhou, Q.	Dual-protein sport supplement and preparation method	*Cocos nucifera* L. *Glycine max* (L.) Merr.	Muscle mass increase	Liquid (tonic)	[103]
57.	Zhou, Q.	A kind of astaxanthin functional sports drink and preparation method	*Glycine max* (L.) Merr. *Linum usitatissimum* L. *Olea europaea* L.	Post-exercise recovery support	Liquid (beverage)	[104]
58.	Zwijsen, R.M.L.	Nutritional compositions for musculoskeletal support for athletes	*Brassica napus* L. *Carthamus tinctorius* L. *Cocos nucifera* L. *Glycine max* (L.) Merr. *Gossypium arboreum* L. *Helianthus annuus* L. *Olea europaea* L. *Zea mays* L.	Nutrition	Liquid (beverage) and bulk-solid (powder)	[105]
